# Clinical and microbiological characteristics of *Nocardia* infections: a 10-Year multicentre study from Türkiye

**DOI:** 10.1007/s10096-026-05470-z

**Published:** 2026-03-25

**Authors:** Eren Ozturk, Elif Aguloglu Bali, Yasemin Cakir Kiymaz, Dilsat Tepe, Ilkay Nur Can, Arda Kaya, Benan Atak Bolataslan, Cemre Bosnak, Birsen Cunetoglu, Fatih Karasin, Hasib Kutay, Mehmet Pekguzel, Merve Buyukkoruk, Semanur Kuzi, Berkan Alp Usta, Yusra Agaoglu, Pelin Irkoren, Murat Aydın, Yesim Uygun Kizmaz, Damla Verendag, Lutfiye Mulazimoglu Durmusoglu, Meliha Cagla Sonmezer, Bedia Mutay Suntur, Hanife Nur Karakoc Parlayan, Selda Sayin Kutlu, Gule Cinar, Kemal Osman Memikoglu, Serap Simsek Yavuz, Sibel Yildiz Kaya, Sila Akhan, Onder Ergonul

**Affiliations:** 1 Faculty of Medicine, Department of Infectious Diseases and Clinical Microbiology, Ankara University, Ankara, Türkiye; 2 Department of Infectious Diseases and Early Warning, Public Health General Directorate, Ankara, Türkiye; 3 Faculty of Medicine, Department of Infectious Diseases and Clinical Microbiology, Sivas Cumhuriyet University, Sivas, Türkiye; 4 Faculty of Medicine, Department of Infectious Diseases and Clinical Microbiology, Karadeniz Technical University, Trabzon, Türkiye; 5 Atakent Hospital, Acıbadem Mehmet Ali Aydınlar University, Istanbul, Türkiye; 6 Faculty of Medicine, Department of Infectious Diseases and Clinical Microbiology, Ege University, Izmir, Türkiye; 7 Pendik Training and Research Hospital, Department of Infectious Diseases and Clinical Microbiology, Marmara University, Istanbul, Türkiye; 8 Faculty of Medicine, Department of Infectious Diseases and Clinical Microbiology, Hacettepe University, Ankara, Türkiye; 9 Adana City Training and Research Hospital, Department of Infectious Diseases and Clinical Microbiology, Adana, Türkiye; 10 Faculty of Medicine, Department of Infectious Diseases and Clinical Microbiology, Kocaeli University , Kocaeli, Türkiye; 11 Faculty of Medicine, Department of Infectious Diseases and Clinical Microbiology, Baskent University, Adana, Türkiye; 12 Faculty of Medicine, Department of Infectious Diseases and Clinical Microbiology, Pamukkale University , Denizli, Türkiye; 13 Soma State Hospital, Manisa, Türkiye; 14 Etlik City Hospital, Department of Infectious Diseases and Clinical Microbiology, Ankara, Türkiye; 15 Faculty of Medicine, Department of Infectious Diseases and Clinical Microbiology, Cerrahpasa University, Istanbul, Türkiye; 16 Faculty of Medicine, Department of Infectious Diseases and Clinical Microbiology, Istanbul University , Istanbul, Türkiye; 17 Unye State Hospital, Ordu, Türkiye; 18 Erzurum Regional Training and Research Hospital, Erzurum, Türkiye; 19 Kosuyolu High Specialization Training and Research Hospital, Istanbul, Türkiye; 20 Faculty of Medicine, Department of Infectious Diseases and Clinical Microbiology, Koc University , Istanbul, Türkiye

**Keywords:** Nocardia, Nocardiosis, Immunocompromise, Treatment, Cerebral Nocardiosis

## Abstract

**Purpose:**

Multicentre studies from countries such as Spain, Australia, Japan, China, and Taiwan have revealed significant geographic variation in *Nocardia* species patterns, resistance profiles, and patient outcomes. In Türkiye, however, most available data originate from single-centre reports with limited species-level identification and antimicrobial resistance profiling, highlighting a significant gap in the current understanding of nocardiosis at the national level. Our aim was to evaluate the clinical and microbiological characteristics of *Nocardia* infections in Türkiye.

**Methods:**

In this multicentre, retrospective observational cohort study, adult patients (≥ 18 years) diagnosed with microbiologically confirmed nocardiosis between January 1, 2014, and December 31, 2024 from 18 tertiary care hospitals in Türkiye were examined.

**Results:**

109 microbiologically confirmed nocardiosis cases were identified, with a mean age of 55.9 years (median 59, range 18–80) and a predominance of male patients (66.1%). The most common presenting symptom was productive cough (40.3%), and sputum was the most frequent specimen type yielding *Nocardia* isolates (41.2%). Species-level identification was achieved in 51 cases, with *Nocardia farcinica* (41.2%), *N. cyriacigeorgica* (29.4%) being the most prevalent species. In multivariate logistic regression, increasing age was independently associated with mortality (OR = 1.053, 95% CI 1.012–1.096, *p* = 0.011) and the CCI was independently associated with mortality (OR = 1.244, 95% CI 1.043–1.483, *p* = 0.015).

**Conclusion:**

This study demonstrates that clinical outcomes in nocardiosis are primarily dictated by patient-intrinsic factors, namely advanced age and cumulative comorbidity burden. This finding requires confirmation in prospective studies.

**Graphical Abstract:**

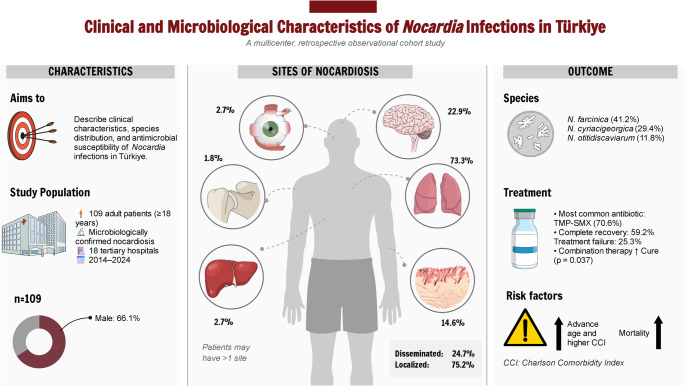

## Introduction

 Nocardiosis is an uncommon but potentially life-threatening infection caused by aerobic, filamentous, partially weakly acid-fast Gram-positive bacteria of the *Nocardia* genus. These organisms are ubiquitous in soil and water and primarily affect immunocompromised individuals, although up to one-third of reported cases occur in immunocompetent hosts [1–4]. Clinical presentations are diverse, ranging from localized cutaneous disease to severe pulmonary involvement and disseminated infections, especially those involving the central nervous system (CNS) and bloodstream. Risk factors include corticosteroid use, solid organ or hematopoietic stem cell transplantation, malignancies, and chronic pulmonary diseases [3–6].

The diagnosis of nocardiosis is often delayed due to its nonspecific clinical presentation and the slow growth of the organism in routine cultures [7, 8]. Understanding the predominant *Nocardia* species and their antimicrobial resistance patterns is therefore essential for effective clinical management. Species differ markedly in pathogenicity and drug susceptibility; for instance, *N. farcinica* is frequently associated with CNS and bloodstream infections and demonstrates broad antimicrobial resistance, while *N. brasiliensis* typically causes cutaneous disease and *N. nova* is often more susceptible to standard therapies [3, 9].

Multicentre studies conducted in countries such as Spain, Australia, Japan, China, and Taiwan have demonstrated significant geographic variations in the patters of *Nocardia* species, their resistance profiles, and patient outcomes [8–12]. In contrast, most of data available in Türkiye, originate from single-centre reports that offer limited species-level identification and antimicrobial resistance profiling. This highlights a significant gap in the current understanding of nocardiosis at the national level [13–17].

To address this gap, we conducted a multicentre retrospective analysis of culture-confirmed nocardiosis cases from tertiary hospitals across Türkiye. Our aim is to evaluate the clinical characteristics, underlying risk factors, species distribution, and antimicrobial susceptibility profiles. To our knowledge, this is the largest national cohort of *Nocardia* infections reported from Türkiye. Our findings provide important insights into regional epidemiology, help inform empirical treatment decisions, and contribute to the global understanding of this complex infection.

## Methods

### Study design and population

This multicentre, retrospective observational cohort study was conducted at 18 tertiary care hospitals in Türkiye. We systematically reviewed the medical records of all adult patients (≥ 18 years) diagnosed with microbiologically confirmed nocardiosis between January 1, 2014, and December 31, 2024. The diagnosis required the isolation of a *Nocardia* species from a clinical specimen, coupled with compatible clinical signs and symptoms of infection. Patients with incomplete critical data (e.g., treatment outcomes) or those without microbiological confirmation were excluded. To prevent overrepresentation, only the first isolate from the same infectious episode was included for each patient. This study was performed in line with the principles of the Declaration of Helsinki and was approved by the Ethics Committee of the Faculty of Medicine at Ankara University (Decision Number: İ01–63-25, Date: January 29, 2025). Site-specific institutional review board approval was also obtained from each participating centre.

## Data collection

A standardized case report form was utilised across all centres to extract data from electronic medical records and patient charts. The data encompassed the following categories: [1] Demographics: age and sex; [2] Clinical Data: comorbidities (assessed using the Charlson Comorbidity Index—CCI), immunosuppressive status, site(s) of infection, clinical presentation, and laboratory values at the time of admission; [3] Microbiological Data: isolated *Nocardia* species, antimicrobial susceptibility profiles, and co-isolated pathogens; [4] Radiological Data: findings from computed tomography (CT), magnetic resonance imaging (MRI), or other relevant imaging modalities; [5] Treatment and Outcomes: initial and final antimicrobial regimens, duration of therapy, reasons for any modification to treatment (e.g., toxicity, treatment failure), and clinical outcomes.

## Diagnostic evaluation

Microbiological identification in each centre followed standard laboratory protocols. Gram staining typically revealed Gram-positive, branching filamentous bacilli, which appeared partially acid-fast with Kinyoun staining. Isolates were cultured on blood agar at 37 °C for up to 7–10 days. Species-level identification was primarily performed using Matrix-Assisted Laser Desorption/Ionization Time-of-Flight Mass Spectrometry (MALDI-TOF MS; Vitek MS V3.0, Biomerieux, France). Antimicrobial susceptibility testing was conducted according to the Clinical and Laboratory Standards Institute (CLSI) M24-A2 guidelines, using methods such as broth microdilution, E-test, or disc diffusion, depending on the local laboratory’s capacity [18]. Co-isolated pathogens were documented when present.

Radiological evaluations were performed as part of standard clinical care. While CT was the primary modality for pulmonary disease, brain imaging (CT or MRI) was performed at the discretion of the treating physician to assess for central nervous system (CNS) involvement. Disseminated infection was defined as the involvement of two or more non-contiguous organs or evidence of hematogenous spread.

## Outcome definitions

The primary outcome was all-cause in-hospital mortality. Secondary outcomes included the clinical response at the end of therapy, which was categorised as follows: [1] Complete Recovery, defined as the complete resolution of all clinical and radiological signs of infection; [2] Partial Recovery, indicating partial improvement in clinical and/or radiological findings; or [3] Treatment Failure, characterised by the persistence or progression of infection, the need for a change in therapy due to a lack of efficacy, or death attributable to nocardiosis. Additionally, any adverse drug reactions that necessitated a modification in treatment were documented.

### Statistical analysis

Descriptive statistics were used to summarise the patient characteristics. Continuous variables were reported as mean ± standard deviation or as median with interquartile range, depending on distribution. Categorical variables were given as counts and percentages. Group comparisons used Student’s t test or the Mann–Whitney U test for continuous variables and chi-square or Fisher’s exact test for categorical variables. In cross tabulations, values are shown as column percentages unless stated otherwise. Two-sided *p* < 0.05 was considered statistically significant.

To identify independent predictors of in-hospital mortality, we built multivariable logistic regression models. Variables with *p* < 0.10 in univariable screening and clinically important covariates were considered for entry, while variables with *p* ≥ 0.60 were excluded. Accordingly, dissemination (*p* = 0.976), immunosuppression (*p* > 0.60), and presence of comorbidity (*p* > 0.60) were not included. TMP SMX use was retained on clinical grounds and because its univariable p was below 0.60, *p* = 0.565. Results are presented as odds ratios with 95% confidence intervals.

Because the Charlson Comorbidity Index incorporates age, entering both together produced notable collinearity. To avoid this, we specified two alternative models. Model A included age and excluded CCI. Model B included CCI and excluded age. In univariable analyses, both age and CCI were associated with mortality. In multivariable analyses, age remained an independent predictor in Model A, and CCI remained an independent predictor in Model B.

Model calibration and discrimination were assessed with the Hosmer–Lemeshow test and the area under the receiver operating characteristic curve. Missing data were handled by complete case analysis. No a priori sample size calculation was performed. Analyses were conducted in IBM SPSS Statistics for Windows, Version 25.0.

## Results

### Demographic and epidemiological characteristics

During the study period, a total of 109 patients with microbiologically confirmed nocardiosis were included from the 18 participating hospitals. The mean age of the patients was 55.89 ± 15.55 years, with a median of 59 years (range: 18–80), and the majority of patients were male (66.1%, *n* = 72). A total of 22.9% (n:25/109) of the patients were living in rural areas, and 30.3% (33/109) had a history of soil exposure. *Nocardia* infection was associated with a previously experienced natural disaster in only three patients. The patients had a median (IQR) Charlson Comorbidity Index of 4 [2–5], indicating a high overall burden of comorbidities. At least one comorbid condition was present in 89% (*n* = 97) of the patients. The most common comorbidities included chronic lung diseases (26.6%, *n* = 29) and hypertension (25.6%, *n* = 28). Among immunosuppressed patients, the leading cause of immunosuppression was corticosteroid use (82.2%, *n* = 60/73), followed by antineoplastic chemotherapy for solid organ malignancies (31.5%, *n* = 23/73) and hematologic malignancies (16.4%, *n* = 12/73). Bone marrow and solid organ transplant recipients constituted 8.2% (*n* = 9) and 7.3% (*n* = 8) of the total cohort, respectively (Table 1).

**Table 1:** Demographic and epidemiological characteristics of 109 patients with nocardiosis

	**n**	**(%)**
**Age**		
Mean ± SD	55.89 ± 15.55	
Median (min-max)	59 (18-80)	
**Sex**		
Male	72	66.1
Female	37	33.9
History of Trauma or Surgery*		
None	79	72.4
Trauma	2	18.3
Surgery	28	25.6
**Exposure factor **		
Natural disaster	3 (2.8%)	104 (97.2%)
Rural area	25 (22.9%)	84 (77.1%)
Soil contact	33 (30.3%)	76 (69.7%)
Comorbidities*		
Chronic lung disease	29	**26.6**
Hypertension	28	25.6
Autoimmune disease	20	18.3
Diabetes mellitus	16	14.6
Chronic renal disease	14	12.8
Haematological malignancy	8	7.3
Solid organ malignancy	7	6.4
Cardiovascular disease	6	5.5
Chronic liver disease	4	3.66
Thyroid dysfunction	3	2.75
No comorbidities	12	11
Other diseases	22	20.1
Types of Chronic Lung Disease*		
COPD	12	**41.3**
Asthma	5	17.2
Bronchiectasis	7	24.1
Lung cancer	9	31
Post-pneumonia sequelae	2	6.8
Interstitial lung disease	7	24.1
Cystic fibrosis	4	13.7
**Immunsuppression Status**		
Yes	73	**67**
No	36	33
Causes of Immunosuppression*		
Chemotherapy for solid organ malignancy	23	31.5
Chemotherapy for haematologic malignancy	12	16.4
HIV infection	4	5.4
Steroid use	60	**82.1**
Treatment for rheumatologic disease	15	20.5
Other causes	11	15
**History of Transplantation**		
Bone marrow transplantation	9	8.2
Solid organ transplantation	8	7.3
History of Pulmonary Infection*		
Pneumonia	49	44.9
Tuberculosis	8	7.3
Fungal infection	6	5.5
Other	12	11

Asterisk (*) indicates that multiple entries per patient may apply. *COPD – Chronic obstructive pulmonary disease HIV – Human immunodeficiency virus

## Clinical presentation and infection sites

The most common presenting-symptoms were productive cough 40.3% (*n* = 44), dry cough 39.4% (*n* = 43), dyspnea 37.6% (*n* = 41), and fever 34.8% (*n* = 38). The mean duration of symptoms before diagnosis was 43 ± 113 days, with a median of 15 days (range: 1–1000 days).

Regarding infection localisation, pulmonary involvement was the most prevalent site, observed in 73.3% (*n* = 80) of cases. CNS involvement was seen in 22.9% (*n* = 25), followed by skin and soft tissue infections (14.6%, *n* = 16). Less frequently affected sites included bloodstream (4.5%), liver (2.7%), bone and joints (1.8%), and ocular involvement (2.7%). Disseminated infections were detected in 24.7% (*n* = 27) of cases (Table 2).

**Table 2:** Clinical characteristics and infection sites

Symptoms*	*n*	(%)
Fever	38	34.8
Dry cough	43	39.4
Sputum production	44	**40.3**
Dyspnea	41	37.6
Chest pain	8	7.3
Confusion	4	3.6
Nausea-vomiting	4	3.6
Headache	7	6.4
Neurological symptoms	16	14.6
Other	31	28.4
Symptom Onset Duration (day)		
Mean ± SD	43 ± 113	
Median (min-max)	15 (1–1000)	
Site of Nocardiosis *		
Lung	80	**73.3**
Eye	3	2.7
Skin and soft tissue	16	14.6
Central nervous system	25	22.9
Bone and joint	2	1.8
Blood	5	4.5
Liver	3	2.7
Other	2	1.8
Type of disease		
Disseminated	27	**24.7**
Localised	82	75.2

*Multiple complaints or infection sites per patient may apply.

### Microbiological findings and laboratory

*Nocardia* species were identified from various clinical specimens, most commonly from sputum (41.2%), abscess material (22.9%), and bronchoalveolar lavage (11.9%). Identification methods included culture (42.2%), MALDI-TOF MS (33.9%), conventional microbiological techniques (12.8%), and PCR (6.4%). Antibiotic susceptibility testing was performed using E-test (69.8%), broth microdilution (23.8%), and disc diffusion method (6.3%).

**Fig. 1:**
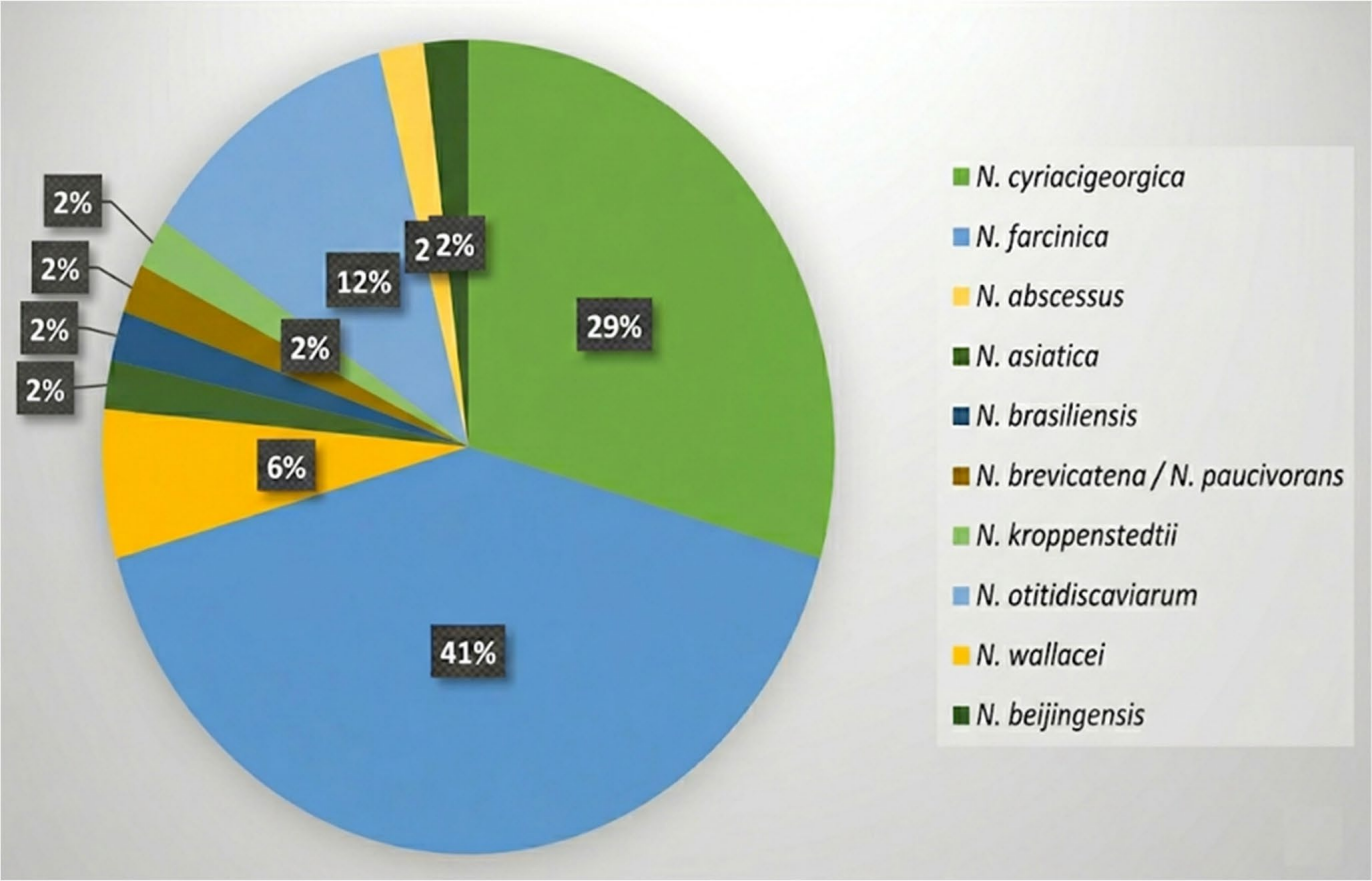
Distribution of *Nocardia* Species Identified in the Study

Species-level identification was achieved in 51 isolates, with *Nocardia farcinica* (41.2%, *n* = 21/51), *N. cyriacigeorgica* (29.4%, *n* = 15/51), and *N. otitidiscaviarum* (11.8%, *n* = 6/51) being the most frequent species (Fig. 1). Gram staining was performed on 69 clinical samples prior to culture, of which 39.1% (*n* = 27/69) were positive for Gram-positive, branching bacilli. Co-infections with other pathogens were common, particularly with gram-negative bacteria (43.4%) and filamentous fungi (23.9%) (Table 3). Antimicrobial susceptibility profiles are detailed in Table 4.

**Table 3:** Specimen Types, Identification, Susceptibility Testing Methods, and Co-pathogens of *Nocardia* Infections

Specimen Type	n	(%)
Sputum	**45**	**41.2**
Bronchoalveolar lavage	13	11.9
Endotracheal aspirate	4	3.6
Blood	9	8.2
Cerebrospinal fluid	3	2.7
Pleural fluid	3	2.7
Conjunctive	2	1.8
Abscess	25	**22.9**
Tissue biopsy	7	6.4
**Identification Methods**		
Conventional	14	12.8
MALDI-TOF	37	**33.9**
PCR	7	6.4
Culture	46	**42.2**
Gram stain-supported additional methods	3	2.7
Phoenix	6	5.5
**Antimicrobial Susceptibility Testing Method**		
Broth microdilution	15	23.8
E-test	44	**69.8**
Disc diffusion	4	6.3
Co-pathogens Detected with Nocardia spp.*		
Actinomyces	4	8.6
Gram-positive bacteria	10	21.7
Gram-negative bacteria	20	**43.4**
Yeast	5	10.8
Mould	11	23.9
Viruses	4	8.6
Mycobacteria	2	1.8

*Multiple co-pathogens may be identified per patient.

**Table 4:** Antimicrobial sensitivity patterns of *Nocardia* isolates*

Antibiotic Tested	% Susceptible	Susceptible/Total (*n*/*N*)	S: *R* Ratio
**TMP-SXT**	79	46/58	46:12
**IMP**	87	35/40	35:5
**AK**	89	34/38	34:4
**CRO**	67	22/33	22:11
**MOX**	89	8/9	8:1
**CIP**	43	17/39	17:22
**LNZ**	85	34/40	34:6
**DOX**	27	3/11	3:8
**CLR**	58	10/17	10:7

*Intermediate results are omitted.TMP-SXT: Trimethoprim–Sulfamethoxazole; IMP: Imipenem; AK: Amikacin; CRO: Ceftriaxone; MOX: Moxifloxacin; CIP: Ciprofloxacin; LNZ: Linezolid; DOX: Doxycycline; CLR: Clarithromycin

### Radiological findings

CT was the most frequently used imaging modality, performed in 79.4% of patients (*n* = 81). MRI was performed in 23 patients (22.5%), primarily for CNS evaluation. Pulmonary imaging was performed in 80 patients. Among patients with pulmonary involvement, the most common radiographic findings were consolidation (54%, *n* = 47) nodules (52.8%, *n* = 46), and cavitary lesions (27.5%, *n* = 24). Bilateral lung involvement was observed in 25.2% (*n* = 22), and multilobar disease in 10.3% (*n* = 9). Brain imaging (CT and/or MRI) was performed in 67 patients, while 32 patients did not undergo brain imaging. Brain imaging revealed solitary lesions in 33.3% (*n* = 24) and multiple lesions in 20.8% (*n* = 15), with bilateral CNS involvement noted in 6.9% (*n* = 5) of patients (Table 5).

**Table 5:** Imaging Methods and Radiological Findings Used in Diagnosis

Method	*n*	(%)
Computed Tomography (CT)	81	**79.4**
Magnetic Resonance Imaging (MRI)	23	22.5
Positron Emission Tomography (PET)	5	4.9
Ultrasonography (USG)	9	8.8
Other	4	3.9
Pulmonary Imaging Findings		
Consolidation	47	**54**
Nodule	46	**52.8**
Cavity	24	27.5
Bronchiectasis	13	14.9
Pneumonic infiltration	11	12.6
Pleural effusion	10	11.4
Single lobe involvement	13	14.9
Multilobar involvement	9	10.3
Bilateral involvement	22	25.2
Cerebral Imaging Findings*		
No suspicious lesion	39	54.1
Solitary lesion	24	33.3
Multiple lesions	15	20.8
Bilateral lesion	5	6.9
No brain imaging performed*	32	29.3

Multiple radiological findings may coexist in the same patient.Percentages for cerebral imaging findings were calculated among patients who underwent brain imaging. The proportion of patients without brain imaging was calculated based on the total study population.

### Treatment Modalities and Prognostic Factors

The most frequently administered antibiotic was trimethoprim-sulfamethoxazole (TMP-SMX), used in 70.6% (*n* = 77) of patients, followed by carbapenems (42.2%) and aminoglycosides (12.8%). In 48% of patients, treatment modifications were implemented, with adverse effects accounting for 34.8% and treatment failure for 25.7% of these adjustments. The mean treatment duration was 121 ± 146 days, with a median of 65 days (range: 1–820 days). The average hospital length of stay was 36 ± 32 days, and the median stay was 30 days (range: 1–176 days). For those requiring intensive care unit (ICU) admission due to nocardiosis, the mean ICU stay was 12 ± 27 days (median: 2 days, range: 0–172 days). Regarding treatment outcomes, 59.2% (*n* = 61) of patients achieved complete recovery, 15.5% (*n* = 16) showed partial recovery, while 25.3% (*n* = 26) of patients experienced treatment failure (Table 6).

**Table 6:** Treatment modalities and outcomes

Antibiotics	n	(%)
TMP-SXT	77	**70.6**
Carbapenem	46	42.2
Aminoglycoside	14	12.8
Quinolone	7	6.4
Linezolid	11	10
Other	35	32.1
Reasons for Treatment Modification		
Treatment failure	11	25.7
Development of adverse effects	15	34.8
Transition to oral sequential therapy	9	20.9
Other	8	18.6
Treatment Durations (days)		
Mean ± SD	121 ± 146	
Median (min-max)	65 (1–820)	
Hospitalisation Duration (days)		
Mean ± SD	36 ± 32	
Median (min-max)	30(1–176)	
ICU Stay Due to Nocardisis		
Mean ± SD	12 ± 27	
Median (min-max)	2(0–172)	
Outcome		
Complete recovery	61	59.2
Partial recovery	16	15.5
Treatment failure	26	25.3

*TMP: trimethoprim–sulphamethoxazole; ICU: intensive care unit*

In univariate analysis, the mean age was significantly higher in the mortality group compared to survivors (61.79 ± 12.64 vs. 52.87 ± 15.87, ***p*** **= 0.003**). A statistically significant association was also observed between CCI and mortality (***p*** **= 0.004**), with higher index scores noted among deceased patients. ICU length of stay did not differ significantly 19.74 ± 36.06 vs. 7.38 ± 19.12 days (*p* = 0.61). No significant between-group differences were observed for TMP treatment, dissemination, presence of comorbidity, immunosuppression, or corticosteroid use (all *p* > 0.05). Full univariate comparisons are shown in Table 7.

Additionally, there was a statistically significant association between treatment modality (monotherapy vs. combination therapy) and cure rates (*p* = 0.037), with higher cure rates observed in patients receiving combination therapy, while those receiving monotherapy had lower cure rates. Combination therapy was more frequently used in patients with diabetes, pulmonary involvement, and positive Gram staining findings (*p* = 0.018, *p* = 0.02, and *p* = 0.037, respectively).

**Table 7:** Factors Associated with Mortality in Univariate Analyses

Variable	Category	Survivors (*n* = 68)	Non-survivors (*n* = 39)	Univariate Analysis (test, *p*)
Age (mean ± SD)		52.87 ± 15.87	61.97 ± 12.64	Mann-Whitney U test, *p* = 0.003
Charlson Comorbidity Index (mean ± SD)		3.23 ± 2.41	4.89 ± 3.48	Mann-Whitney U test, *p* = 0.004
Duration of ICU stay (days, mean ± SD)		7.38 ± 19.12	19.74 ± 36.06	Mann-Whitney U test, *p* = 0.61
TMP treatment, n (%)*	Given	47 (69.1)	10 (25.6)	χ² test, *p* = 0.565
	Not given	21 (30.9)	29 (74.6)	
Dissemination, n (%)*	With	17 (25.4)	10 (25.6)	χ² test, *p* = 0.976
	Without	50 (74.6)	29 (74.4)	
Presence of comorbidity, n (%)*	Yes	59 (86.8)	35 (89.7)	χ² test, *p* = 0.765
	No	9 (13.2)	4 (10.3)	
Immunosuppression, n (%)*	Present	45 (66.2)	27 (69.2)	χ² test, *p* = 0.832
	Absent	23 (33.8)	12 (30.8)	
Corticosteroid use, n (%)*	Yes	40 (62.5)	20 (51.3)	χ² test, *p* = 0.306
	No	24 (37.5)	19 (48.7)	

*Values are expressed as column percentages.TMP: trimethoprim–sulphamethoxazole; ICU: intensive care unit

In multivariate logistic regression, two models were specified to address collinearity between age and CCI. In Model A (age included, CCI excluded), increasing age was independently associated with mortality (OR = 1.053, 95% CI 1.012–1.096, *p* = 0.011). In Model B (CCI included, age excluded), the CCI was independently associated with mortality (OR = 1.244, 95% CI 1.043–1.483, *p* = 0.015). Other covariates, including TMP-SMX use (*p* = 0.403 and *p* = 0.401), ICU length of stay (*p* = 0.176 and *p* = 0.056), corticosteroid use (*p* = 0.579 and *p* = 0.409), dissemination (*p* = 0.903), presence of comorbidities (*p* = 0.825), and immunosuppression (*p* = 0.340), were not significant predictors of mortality. Although corticosteroid use showed a non-significant trend toward increased mortality (Model A OR = 1.350, 95% CI 0.468–3.897; Model B OR = 1.559, 95% CI 0.544–4.471), this did not reach statistical significance. Both models demonstrated good calibration (Model A: Hosmer–Lemeshow χ²=6.298, *p* = 0.614; Model B: χ²=5.936, *p* = 0.654) (Tables 8 and 9).

**Table 8:** Multivariate analysis of mortality predictors – Model A (Age only)

Model A			
Variable	Multivariate Analysis		
	OR	[95% CI]	*p*
TMP treatment	1.626	0.520–5.079	0,403
ICU length of stay	1.016	0.993–1.041	0.176
Corticosteroid use	1.350	0.468–3.897	0,579
Age	1.053	1.012–1.096	**0**,**011**

TMP:trimethoprim–sulfamethoxazole; ICU: intensive care unit

**Table 9:** Multivariate analysis of mortality predictors – Model B (CCI only)

Model B			
Variable	Multivariate Analysis		
	OR	[95% CI]	*p*
TMP treatment	1.642	0.516–5.219	0,401
ICU length of stay	1.025	0.999–1.050	0,056
Corticosteroid use	1.559	0.544–4.471	0,409
Charlson Comorbidity Index	1.244	1.043–1.244	**0**,**015**

TMP: trimethoprim–sulphamethoxazole; ICU: intensive care unit

## Discussion

In this extensive, multicentre study of nocardiosis conducted in Türkiye, we identified host-related factors specifically advanced age and a significant comorbidity burden as the primary independent predictors of in-hospital mortality. Notably, disease-specific characteristics such as dissemination or immunosuppression status, while clinically relevant, did not show independent association with outcomes in our multivariable models. This suggests that the prognosis in nocardiosis is driven less by the aggressiveness of the infection itself and more by the patient’s underlying vulnerability. The CCI, which reflects the cumulative burden of chronic diseases, emerged as a robust prognostic marker, underscoring the necessity for aggressive management in frail, comorbid patients.

To our knowledge, this study represents the most extensive investigation of *Nocardia* infections in Türkiye to date. A key finding is the apparent increase in incidence over the last decade, a trend consistent with reports from other regions [4, 19, 20]. This rise is likely multifactorial, reflecting not only a true increase due to the expanding population of immunosuppressed individuals but also enhanced diagnostic capabilities, particularly the widespread adoption of MALDI-TOF MS in tertiary care centres. The concentration of cases within these specialized hospitals, which manage complex patient populations in oncology, hematology, and transplantation units, further highlights nocardiosis as a significant emerging opportunistic infection in modern healthcare settings.

The microbiological landscape of nocardiosis in our cohort aligns partially with previous regional reports, with *N. farcinica* and *N. cyriacigeorgica* remaining the predominant species [13, 14, 17, 21]. However, we also report the first identification of *Nocardia kroppenstedtii* in Türkiye, a finding that underscores the evolving epidemiology of these pathogens and the critical importance of species-level identification. Given that different *Nocardia* species exhibit distinct antimicrobial susceptibility profiles, ongoing surveillance is essential to inform empirical treatment guidelines and track the emergence of rare or resistant species [10].

Consistent with the global literature, our cohort showed a male predominance and identified chronic lung disease and long-term corticosteroid use as the most significant predisposing factors [6, 22]. The proportion of patients receiving corticosteroids in our study was notably high compared to international cohorts [3, 6, 12, 19], potentially reflecting regional therapeutic practices. Although corticosteroid use did not emerge as an independent predictor of mortality, likely due to insufficient statistical power, its role as a key risk factor for acquiring the infection is undeniable.

The clinical presentation in our study underscores the diagnostic challenges of nocardiosis. With pulmonary disease being the most common manifestation, its non-specific radiological findings—such as consolidation, nodules, and cavities—often mimic more common conditions like tuberculosis or invasive fungal infections, leading to significant diagnostic delays [4, 23]. The median time to diagnosis in our cohort was 43 days, a delay that can allow for disease progression. Furthermore, the rate of CNS involvement observed in our cohort (22.9%), a site frequently associated with poor outcomes, supports the recommendation for routine contrast-enhanced brain imaging in all patients with nocardiosis, even in the absence of neurological symptoms, in order to prevent unrecognised involvement and potential treatment failure [1]. Limited use of brain imaging may lead to an underestimation of the true frequency of CNS involvement.

Regarding therapy, TMP-SMX remains the cornerstone of treatment. However, our finding that combination therapy was associated with higher cure rates than monotherapy warrants careful interpretation. This observation is susceptible to confounding by indication, as clinicians may have preferentially administered combination regimens to patients with more severe disease. Therefore, while our data supports the common practice of using multi-drug regimens for severe or disseminated nocardiosis, it does not establish causality. This highlights a critical knowledge gap and underscores the urgent need for prospective, randomised controlled trials to define optimal therapeutic strategies and durations of treatment [1, 7, 24]. The study contributes to empirical treatment strategies and highlights a rising public health threat in immunosuppressed patients.

This study has several inherent limitations. Its retrospective nature is subject to information bias and missing data. Diagnostic protocols and antimicrobial susceptibility testing methods varied across the 18 centres over the 10-year period, introducing potential heterogeneity. Species-level identification was not available for nearly half of the isolates, which could introduce a bias in the observed species distribution. Furthermore, the lack of molecular methods precluded a more granular analysis of species identification and resistance mechanisms. Despite these limitations, the study’s strength lies in its large, multicentre design, which provides the most robust and generalisable data on nocardiosis from this region to date.

In conclusion, this study demonstrates that clinical outcomes in nocardiosis are primarily dictated by patient-intrinsic factors, namely advanced age and cumulative comorbidity burden. Our findings also highlight nocardiosis as an emerging public health challenge in Türkiye, driven by an expanding high-risk patient population and improved diagnostics. While combination antimicrobial therapy was associated with better outcomes, this finding requires confirmation in prospective studies. Clinicians should maintain a high index of suspicion for nocardiosis in immunocompromised patients with compatible syndromes and consider routine brain imaging to rule out occult CNS disease.

## Data Availability

The datasets analyzed during the current study are available from the corresponding author upon reasonable request.
